# The Bioenergetic Status Relates to Dopamine Neuron Loss in Familial PD with PINK1 Mutations

**DOI:** 10.1371/journal.pone.0051308

**Published:** 2012-12-10

**Authors:** Rüediger Hilker, Ulrich Pilatus, Carsten Eggers, Johann Hagenah, Julia Roggendorf, Simon Baudrexel, Johannes C. Klein, Bernd Neumaier, Gereon R. Fink, Helmuth Steinmetz, Christine Klein, Elke Hattingen

**Affiliations:** 1 Department of Neurology, Goethe-University, Frankfurt/Main, Germany; 2 Institute for Neuroradiology, Goethe-University, Frankfurt/Main, Germany; 3 Department of Neurology, University of Cologne, Cologne, Germany; 4 Section of Clinical and Molecular Neurogenetics at the Department of Neurology, University of Lübeck, Lübeck, Germany; 5 Max-Planck-Institute for Neurological Research, Cologne, Germany; The University of Chicago, United States of America

## Abstract

Mutations in the *PINK1* gene cause autosomal recessive familial Parkinson’s disease (PD). The gene encodes a mitochondrial protein kinase that plays an important role in maintaining mitochondrial function and integrity. However, the pathophysiological link between mutation-related bioenergetic deficits and the degenerative process in dopaminergic neurons remains to be elucidated. We performed phosphorous (^31^P) and proton (^1^H) 3-T magnetic resonance spectroscopic imaging (MRSI) in 11 members of a German family with hereditary PD due to *PINK1* mutations (PARK6) compared to 23 age-matched controls. All family members had prior 18-Fluorodopa (FDOPA) positron emission tomography (PET). The striatal FDOPA uptake was correlated with quantified metabolic brain mapping in MRSI. At group level, the heterozygous *PINK1* mutation carriers did not show any MRSI abnormalities relative to controls. In contrast, homozygous individuals with manifest PD had putaminal GPC, PCr, HEP and β-ATP levels well above the 2SD range of controls. Across all subjects, the FDOPA K_i_ values correlated positively with MI (r = 0.879, p<0.001) and inversely with β-ATP (r = −0.784, p = 0.008) and GPC concentrations (r = −0.651, p = 0.030) in the putamen. Our combined imaging data suggest that the dopaminergic deficit in this family with PD due to PINK1 mutations relates to osmolyte dysregulation, while the delivery of high energy phosphates was preserved. Our results corroborate the hypothesis that PINK1 mutations result in reduced neuronal survival, most likely due to impaired cellular stress resistance.

## Introduction

Mutations of the *PTEN-induced putative kinase 1 (PINK1*) gene mapped to chromosome 1p36 (PARK6) were reported in up to 5% of patients with autosomal recessive Parkinson’s disease (PD) [1], [2]. PINK1 encodes a mitochondrial serine-threonine kinase, which is involved in the protection of neurons against cellular stress conditions [3], [4] and in maintaining mitochrondrial function and morphology [5]. Recent studies on fibroblasts from PD patients with endogenous expression of mutant PINK1 pointed to respiratory chain deficiencies and enhanced production of reactive oxygen species (ROS), but yielded heterogenous data on cellular ATP levels, activity of respiratory chain enzymes and antioxidant defense mechanisms [6], [7], [8], [9].

In order to investigate the pathophysiology of PINK1-associated PD in a large German family with a PINK1 nonsense mutation (c.1366C>T; p.Q456X), we performed combined phosphorous (^31^P) and proton (^1^H) magnetic resonance spectroscopic imaging (MRSI) on a 3-Tesla scanner in 11 family members who already had participated in a prior study with 18-Fluorodopa (FDOPA) positron emission tomography (PET) [10]. The graded severity of striatal FDOPA loss in this cohort with either a single or two mutated *PINK1* alleles allowed for correlating individual findings of metabolic brain mapping in MRSI with previously obtained PET data. We measured adenosine triphosphate (ATP) as the final energy acceptor generated by the mitochondrial electron transport system, the short-term energy store phosphocreatine (PCr), the glial activity and osmotic status marker myo-inositol (MI), and phosphomono- (PME) and -diesters (PDE) as representative components of neuronal membranes.

**Table 1:** ^31^P MRSI data of the PINK1 study cohort compared to age-matched healthy controls.

		controls (n = 23)	95%CI	PINK1 heterozygous (n = 9)	95%CI	PINK1 homozygous patient II.5	PINK1 homozygous = patient II.7
**PEth** [mmol/l]	mesencephalon	1.12 (0.17)	1.05–1.21	1.10 (0.13)	0.94–1.27	1.38	1.46
	putamen	1.04 (0.09)	1.00–1.07	0.97 (0.11)	0.89–1.06	1.20	1.18
**GPE** [mmol/l]	mesencephalon	1.40 (0.28)	1.27–1.55	1.57 (0.26)	1.32–1.88	1.62	1.88
	putamen	1.31 (0.16)	1.25–1.38	1.30 (0.15)	1.19–1.42	1.78*	2.02*
**GPC** [mmol/l]	mesencephalon	1.44 (0.17)	1.38–1.54	1.47 (0.12)	1.32–1.62	1.60	2.02
	putamen	1.54 (0.10)	1.49–1.59	1.54 (0.08)	1.48–1.59	1.85*	1.75*
**PCr** [mmol/l]	mesencephalon	3.22 (0.35)	3.08–3.41	3.20 (0.26)	2.99–3.43	3.25	3.75
	putamen	2.99 (0.19)	2.91–3.08	2.85 (0.24)	2.66–3.04	3.49*	3.38*
**β-ATP** [mmol/l]	mesencephalon	1.24 (0.29)	1.11–1.40	1.23 (0.16)	1.02–1.43	1.82	1.42
	putamen	1.79 (0.20)	1.69–1.88	1.66 (0.12)	1.57–1.76	2.01	2.22*
**Pi** [mmol/l]	mesencephalon	1.08 (0.16)	1.01–1.17	0.94 (0.19)	0.84–1.09	0.85	1.38
	putamen	1.08 (0.13)	1.02–1.13	0.96 (0.11)	0.87–1.05	1.15	1.13
**PCho** [mmol/l]	mesencephalon	0.19 (0.07)	0.17–0.22	0.21 (0.03)	0.17–0.24	0.11	0.25
	putamen	0.17 (0.04)	0.16–0.19	0.15 (0.04)	0.12–0.18	0.16	0.26*
**HEP** [mmol/l]	mesencephalon	4.46 (0.44)	4.28–4.71	4.43 (0.27)	4.21–4.66	5.07	5.17
	putamen	4.78 (0.36)	4.63–4.94	4.52 (0.29)	4.29–4.74	5.50*	5.60*
**LEP** [mmol/l]	mesencephalon	5.47 (1.14)	4.93–6.00	5.28 (1.26)	4.96–6.60	3.72	4.20
	putamen	7.44 (0.69)	7.14–7.74	7.61 (1.06)	6.79–8.42	6.01*	6.36

Data are given as mean (1SD). 95% CI = 95% confidence interval. Bold data with an asterisk mark values outside of the 2SD range of controls.

**Table 2:** ^1^H MRSI data of the PINK1 study cohort compared to age-matched healthy controls.

		controls(n = 23)	95%CI	PINK1 heterozygous (n = 9)	95%CI	PINK1 homozygous patient II.5	PINK1 homozygous patient II.7
**MI** [mmol/l]	mesencephalon	7.25 (1.10)	6.73–7.77	7.57 (0.97)	6.32–8.10	5.29	5.97
	putamen	7.38 (0.94)	6.98–7.79	7.50 (0.85)	6.85–8.15	5.66	6.23
**tCho** [mmol/l]	mesencephalon	2.93 (0.42)	2.72–3.13	2.79 (0.38)	2.36–3.24	2.46	2.38
	putamen	2.98 (0.37)	2.82–3.13	3.00 (0.41)	2.69–3.32	2.89	2.97
**tCre** [mmol/l]	mesencephalon	7.60 (1.02)	7.12–8.08	7.33 (1.07)	6.27–8.74	6.11	6.57
	putamen	9.31 (0.70)	9.01–9.61	9.45 (1.00)	8.68–10.21	7.98	8.56
**NAA** [mmol/l]	mesencephalon	14.96 (1.83)	14.11–15.82	13.71 (2.04)	11.55–16.45	13.48	13.39
	putamen	13.95 (1.26)	13.40–14.50	13.20 (1.11)	12.33–14.05	11.96	12.78
**Glx** [mmol/l]	mesencephalon	9.59 (3.45)	7.97–11.20	10.21 (1.76)	7.95–12.07	12.42	12.18
	putamen	11.28 (1.78)	10.51–12.04	11.64 (1.35)	10.60–12.68	8.36	8.45

Data are given as mean (1SD). 95% CI = 95% confidence interval.

**Table 3:** Calculated MRSI data of the PINK1 study cohort compared to age-matched healthy controls.

		controls(n = 23)	95%CI	PINK1 heterozygous(n = 9)	95%CI	PINK1 homozygous patient II.5	PINK1 homozygous patient II.7
**rCho** [mmol/l]	mesencephalon	0.39 (0.34)	0.24–0.55	0.31 (0.28)	0.03–0.60	0.008	0.001
	putamen	0.37 (0.28)	0.25–0.49	0.42 (0.29)	0.19–0.64	0.013	0.067
**Cre** [mmol/l]	mesencephalon	2.08 (0.89)	1.66–2.49	1.97 (0.75)	1.20–2.88	1.03	0.84
	putamen	3.52 (0.47)	3.32–3.72	3.76 (0.74)	3.19–4.33	2.09*	2.61
**ADP** [mmol/l]	mesencephalon	0.02 (0.01)	0.02–0.03	0.19 (0.01)	0.01–0.03	0.14	0.008
	putamen	0.05 (0.01)	0.05–0.06	0.06 (0.01)	0.05–0.07	0.03*	0.04

Data are given as mean (1SD). 95% CI = 95% confidence interval. Bold data with an asterisk mark values outside of the 2SD range of controls.

## Methods

### Standard Protocol Approval, Registration and Patient Consent

The study was approved by the Ethics Committee of the Faculty of Medicine at the University of Frankfurt/Main (364/08) in accordance with the declaration of Helsinki. All participants gave written informed consent. In a thorough examination by an experienced neurologist, none of the study subjects had cognitive dysfunction or dementia which might have compromised their capacity to agree with participation.

### Study Subjects

Eleven members of Family W with *PINK1* missense mutations were included. Two women with homozygous mutations (II.5 and II.7 of pedigree of family W [10]) had typical levodopa-responsive PD (patient II.5: age 72 years, disease duration 11 years, Unified Parkinson’s disease Rating Scale (UPDRS-III) [11] off- medication 20, Hoehn and Yahr Scale (HY) [12] II; patient II.7∶65 years, disease duration 12 years, UPDRS III off-medication 21, HY II). Nine heterozygous mutation carriers (7 men, 2 women, mean age 47.1±6.0 years) also participated. Of these, seven had mild bradykinesia, resting tremor or reduced arm swing during walking (mean UPDRS III medication-off 4±4), two men presented without clinical signs. All study participants had undergone FDOPA-PET 30 months prior to MRSI whose results were published in a recent paper [10]. However, two family members with homozygous PINK1 mutations who also attended the PET study were not able to take part in the current MRSI study. Twenty-three age- and gender-matched healthy individuals (6 men, 17 women; mean age 46.0±9.6 years) without neurological, psychiatric or systemic disease served as control group for MRSI.

**Figure 1:**
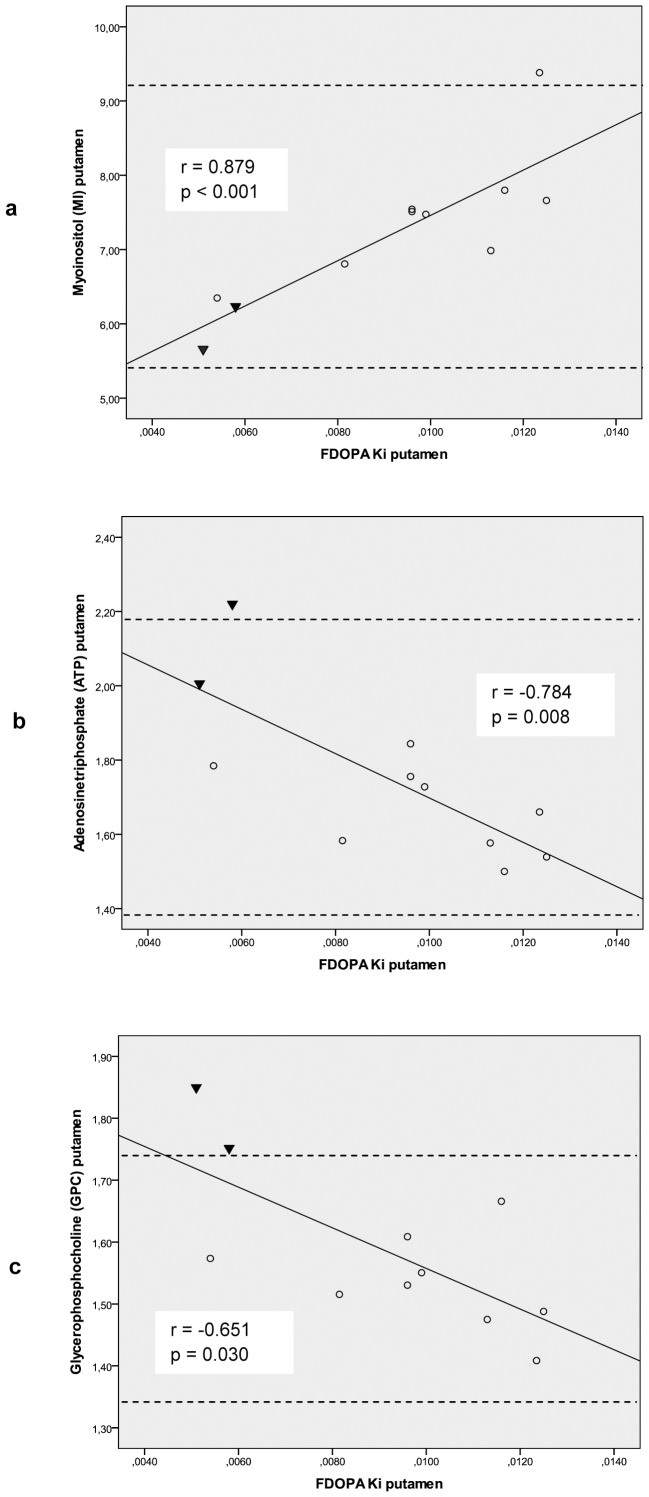
Correlation of MRSI and PET findings in the putamen. Across the entire study cohort with either a single heterozygous (n = 9, open circles) or two homozygous PINK1 mutations (n = 2, black triangles), the mean putaminal FDOPA K_i_ values (min^−1^) determined by PET correlated positively with MRSI-measured MI levels (r = 0.879, p<0.001) (a), but inversely with ATP (r = −0.784, p = 0.008) (b) and GPC concentrations (r = −0.651, p = 0.030) (c). Spearman rank correlation, p-values give the significance level. The dashed horizontal lines set the upper and lower limits of the 2SD range in healthy controls. Note that we found concentrations above the upper limit for GPC in both and for ATP in one of the homozygous mutation carriers.

**Table 4:** Correlation of striatal FDOPA-PET K_i_ values with selected putaminal MRSI data given for all PINK1 mutation carriers (n = 11) and for individuals with heterozygous mutations only (n = 9).

		MRSI putamen					
		MI		GPC		β-ATP	
		Homo- andHeterozygoteindividuals(n = 11)	Heterozygoteindividuals (n = 9)	Homo- andHeterozygoteindividuals(n = 11)	Heterozygoteindividuals (n = 9)	Homo- andHeterozygoteindividuals(n = 11)	Heterozygoteindividuals (n = 9)
**FDOPA K_i_**	**caudate**	r = ,683 p = 0,020	n.s.	r = −,692 p = 0,018	n.s.	r = −,747 p = 0,008	r = −,686 p = 0,041
	**putamen**	r = ,879 p<0,001	r = ,795 p = 0,01	r = −,651 p = 0,030	n.s.	r = −,784 p = 0,008	r = −,669 p = 0,049
	**putamen anterior part**	r = ,843 p = 0,001	r = ,762 p = 0,017	r = −,738 p = 0,010	n.s.	r = −,743 p = 0,009	n.s.
	**putamen posterior part**	r = ,891 p<0,001	r = ,817 p = 0,007	n.s.	n.s.	r = −,773 p = 0,005	n.s.

r = Spearman rank correlation coefficient; n.s. = not significant.

### FDOPA Positron Emission Tomography (PET)

FDOPA-PET scans were performed at the Max-Planck Institute for Neurological Research, Cologne, Germany according to standard methods published recently [10].

### Magnetic Resonance Spectroscopy Imaging (MRSI)

MRSI was undertaken at the Brain Imaging Center of Goethe University Frankfurt/Main on a 3 Tesla whole-body system (Magnetom Trio, Siemens Medical AG, Erlangen, Germany) with a double-tuned ^1^H/^31^P volume head coil (Rapid Biomedical, Würzburg, Germany). A combination of point resolved selective spectroscopy and outer volume suppression was used to select volumes-of-interest in ^1^H MRSI (TR 1500 ms, TE 30 ms, 2 acquisitions, 240×240 mm^2^ FOV). We obtained midbrain 2D ^1^H MRSI by a coronal slice aligned to the dorsal border of the pontine and midbrain tegmentum (28×28 matrix extrapolated to 48×48, 10 mm slice thickness). A second axial ^1^H MRSI covered the putamen (16×16 matrix extrapolated to 32×32; 12 mm slice thickness) [13]. Proton decoupling during ^31^P data acquisition (TR 2000 ms, TE 2.3 ms, 10 acquisitions, flip angle of 60°, 10×10×8 matrix extrapolated to 20×20×16, 300×300×200 mm^3^ FOV) was achieved by wideband alternating phase low power technique for zero residual splitting (WALTZ4).

### MRSI Data Processing

Partial volume effects originating from the cerebrospinal fluid were taken into account by the fraction of grey and white matter taken from isotropic T1 weighted 3D images of the total brain [14]. These were aligned to the MRSI slab followed by digital filtering to mimic the effect of poor point spread function and resolution caused by the limited number of phase encoding steps. [15] The protocol was previously described in detail [13]. The resulting parameter maps provided values for the partial volume of grey and white matter for each voxel. ^1^H MRSI spectra were fitted with the software tool LC Model (Provencher, http://s-provencher.com). Baseline correction was performed including macromolecules. ^31^P spectra were analyzed in the time domain with the jMRUI software tool (Version 3.0, http://www.mrui.uab.es) employing a non-linear least square fitting algorithm (AMARES). Absolute concentrations were calculated by referring to independent measurements with a spherical phantom containing a solution of 100 mmol/l acetate as calibration standard for ^1^H data (TR = 10 s) and 20 mmol/l phosphate for ^31^P data (TR = 60 s). For both phantoms, coil loading was within the 20% range of those used in patient scans. Corrections for T1 and T2 relaxations were performed as described previously [13], [16]. T2 correction was not performed for ^31^P data. Volume concentrations were converted into tissue concentrations by division with the tissue fraction (sum of grey and white matter), i.e. assuming no metabolites in the CSF fraction.

### 31P MRSI Metabolites

The phosphomonoesters phosphocholine (PCho) and phosphoethanolamine (PEth) are precursors of neuronal membrane lipids, whereas the phosphodiesters glycerophosphocholine (GPC) and glycerophosphoethanolamine (GPE) represent membrane lipid catabolites. Hence, the relation of these metabolites provides information on the balance between membrane constitution and degradation. ATP is the end product of oxidative phosphorylation. ATP exhibits 3 signals according to the α-(7.5 ppm,) β-(16.5 ppm), and γ-(2.5 ppm) phosphate group. While the α-and γ-signals coincide with the α-and β-signals from ADP, the β-ATP signal is specific for ATP and its intensity can be considered as a marker for total ATP concentration. Conversion of phosphocreatine (PCr) to creatine (Cre) and inorganic phosphate (Pi) via the creatine kinase reaction represents the most important short term energy buffer of high-energy phosphates (HEP).

### ^1^H MRSI Metabolites

Pooled choline-containing compounds (tCho) are a marker for membrane related processes. tCho represents the sum of the phosphorylated compounds PCho and GPC and an additional choline fraction which cannot be detected by ^31^P MRS (rCho). N-acetyl-aspartate (NAA) is formed in neuronal mitochondria and also considered as a marker of mitochondrial integrity and function in neurons. Pooled creatine compounds (tCre) represent the sum of unphosphorylated Cr and phosphocreatine (PCr). The signal pattern Glx is attributed to the sum of glutamate (Glu) and glutamine (Gln) signals. Glutamate is the most important excitatory neurotransmitter of the brain. Myoinositol (MI) is regarded to be a glial marker serving as an important osmolyte in regulating the cell volume of astrocytes.

### Calculated Parameters

ADP and Cr levels were calculated from the local tissue concentrations. The residual signal resonating at 3.2 ppm in the ^1^H spectra was calculated as:

[rCho] = [tCho] – ([PCho]+[GPC]).

Further calculations were made for:

High-energy phosphates: [HEP] = [β-ATP]+[PCr].

Low-energy metabolites: [LEP] = [ADP]+([tCr] − [PCr])+[Pi].

### Statistics

Statistical analysis was performed with STATISTICA (version 7.1, StatSoft, Tulsa, OK, USA) and SPSS 19.0 for Windows (IBM, Chicago, IL, USA, **Error! Hyperlink reference not valid.** MRSI metabolites and FDOPA K_i_, values were averaged for both cerebral hemispheres. We tested for between-group differences using the non-parametric Mann-Whitney-U-Test. Moreover, we calculated Spearman rank correlation of FDOPA K_i_ and MRSI data. For all tests, the significance threshold was set at p<0.05 (two-tailed).

## Results

### FDOPA PET

The mean FDOPA K_i_ values of the nine heterozygous mutation carriers were 0.0103±0.0023 min^−1^ (caudate), 0.0100±0.0023 min^−1^ (entire putamen), 0.0104±0.0023 min^−1^ (anterior putamen) and 0.0094±0.0023 min^−1^ (posterior putamen). Both homozygous PD patients (II.5 and II.7 of pedigree of family W [10]) had severely reduced FDOPA K_i_ levels: 0.0090 min^−1^ and 0.0057 min^−1^ (caudate), 0.0051 min^−1^ and 0.0058 min^−1^ (entire putamen), 0.0070 min^−1^ and 0.0072 min^−1^ (anterior putamen), and 0.0036 min^−1^ and 0.0046 min^−1^ (posterior putamen). For comparison, reference K_i_ data of healthy controls obtained in our PET laboratory [10] amount to 0.0122±0.0014 min^−1^ (caudate), 0.0127±0.0014 min^−1^ (entire putamen), 0.0132±0.0013 min^−1^ (anterior putamen) and 0.0123±0.0015 min^−1^ (posterior putamen).

### MRSI

MRSI did not reveal any significant mean differences between heterozygous PINK1 mutation carrriers and age-matched controls (Tables 1–3). Both homozygous women with clinically manifest PD had putaminal peak levels of the membrane catabolites GPC and GPE and of the bioenergy marker HEP above the controls’ 2SD range (Table 1). With respect to single HEP components, patient II.7 revealed both higher β-ATP and PCr, whereas patient II.5 had higher PCr only. Moreover, patient II.7 showed increased PCho and patient II.5 decreased LEP, Cre and ADP levels in the putamen.

### Correlation of PET and MRSI Data

Over the entire study cohort (n = 11), the mean FDOPA K_i_ values correlated positively with MI levels (r = 0.879, p<0.001), and inversely with β-ATP (r = −0.784, p = 0.008) and GPC concentrations (r = −0.651, p = 0.030) in the putamen (Table 4 and Figure 1a–c). Analyzing heterozygote mutation carriers alone (n = 9), appropriate correlations were found for MI and β-ATP, but not for GPC (Table 4).

## Discussion

The MRSI findings in heterozygous family members with a mild dopaminergic dysfunction in FDOPA PET suggest that **heterozygous loss-of-function** is associated with a normal level of high-energy phosphates in mesostriatal brain tissue. In contrast, two homozygous PINK1 mutation carriers with typical PD phenotype and severely reduced FDOPA uptake revealed pathological concentrations of putaminal MRSI metabolites. In particular, elevated levels of the membrane breakdown product GPC and of the high energy phosphates PCr and β-ATP were observed. Across all family members, the putaminal FDOPA K_i_ values correlated inversely with β-ATP and GPC levels and positively with MI concentrations. Our data suggest that dopaminergic dysfunction in this family with PINK1 mutations is accompanied by unaffected delivery of high-energy phosphates and osmolyte dysregulation in the striatum.

PINK1 predominantly localizes to mitochondria [17] indicating that the protein plays a role in neuronal energy metabolism. It is also involved in cell signaling and intracellular Ca^2+^ homeostasis [18]. In mammalian cells with PINK1 knockdown, disturbed energy maintenance with increased ATP consumption has been found in case of high-energy demand [6], [19]. Thus, it has been argued that PINK1-deficient neurons are unable to adequately fuel several ATP-demanding processes. However, our in vivo brain imaging data in family W indicate that either homo- or heterozygous carriers of a PINK1 mutation do not inevitably reveal a noticeable depletion of high-energy phosphates in the striatum. Therefore, it is possible that PINK1 mutations in family W do not induce relevant changes of the bioenergetic status in affected neurons. In line with this interpretation, recent in-vitro studies investigating the impact of PINK1 mutations on mitochondrial ATP production did not reveal uniform evidence for the failure of energy supply in these cells. Whereas the complexes of the mitochondrial oxidative phosphorylation (OXPHOS) system (respiratory chain complexes I-IV finally yielding ATP via complex V) appeared normal in most PINK1-deficient cells [7], [9], [18], [19], another group reported diminished enzyme activities [20]. ATP steady-state levels were decreased in fibroblasts with homozygous PINK1 nonsense mutations (p.Q456X), but unaffected in those harbouring a homozygous missense mutation (p.V170G) [7].

Unexpectedly, we even found higher putaminal PCr and HEP levels in both PD patients with homozygous PINK1 mutations compared to controls. Due to the small number of homozygous study participants, the conclusions allowed to draw from these data are surely limited, and it cannot be excluded with certainty that these findings in two individuals are spurious. However, considering the strong inverse correlation between striatal dopaminergic degeneration and β-ATP levels over the entire study cohort, we alternatively hypothesize that the increased levels of high-energy phosphates in homozygous PINK1 are accomplished by ATP generation beyond the OXPHOS system, possibly at the expense of intracellular milieu changes. Some recent in vitro findings corroborate this hypothesis: While skin fibroblasts of a patient with a homozygous PINK1 mutation showed lowered ATP levels in a galactose medium, higher ATP concentrations were observed in a glucose solution presumably due to enhanced glycolytic activity [21]. The authors concluded that depressed OXPHOS activity was counterbalanced by an increased ATP synthesis via glycolysis potentially inducing a noxious lactate acidosis. In this regard, it is important to note that in-vivo MRSI mapping includes the whole brain tissue with neurons and surrounding neuroglia, which is a key advantage of in-vivo brain imaging compared to the somewhat artificial study conditions in isolated cell cultures. Whereas neurons failed to significantly raise glycolysis after pharmacological blocking of respiration [22], brain tissue is able to utilize enhanced glycolysis in astrocytes to maintain mitochondrial respiration and ATP levels in case of increased energy demand [23]. Astrocytes were shown to participate directly in transmitter processing, osmotic regulation and in substrate provision for adjacent neurons [24]. Another line of evidence suggests that the limitation of mitochondrial OXPHOS substrates seems to be a key mechanism in PINK1 pathology, which may result in a switch from production to hydrolysis of ATP to ADP via complex V [6]. This phenomenon was reversed by adding respiratory chain substrates pointing to compensatory mechanisms directed at the maintenance of the mitochondrial membrane potential and of sufficient ATP levels in PINK1 deficient cells. In line with this hypothesis, complex II and/or III activity was elevated in fibroblasts of a further patient with a homozygous PINK1 missense mutation [7].

Furthermore, we found a close correlation of decreased striatal MI levels with low FDOPA K_i_ in the entire study cohort, which persisted in the putamen when heterozygote family members were analyzed separately. Therefore, the measurements of the two homozygous PINK1 mutation carriers are not skewing the correlation towards positive in this case, which suggests a true relationship between putaminal dopamine neuron loss and MI concentrations. MI is a sugar alcohol with a molecular structure similar to glucose. The molecule is predominantly found in astrocytes and, therefore, often interpreted as a gliosis marker [25]. However, MI is also a potent osmoregulator and an intracellular messenger molecule [26]. Increased cerebral MI concentrations have been reported in patients with AD and mild cognitive impairment [27], [28]. A previous MRSI study in two siblings with a homozygous PINK1 mutation outside of the kinase domain found elevated MI levels in the basal ganglia [29]. In contrast, MI reductions are more difficult to interpret given the complex biochemical pathways in which this molecule is involved. The cell volume of neurons faced with osmotic stress is long-term regulated by intracellular concentration changes of organic solutes, such as inositol, betaine and amino acids [30]. In a previous ^31^P-MRSI study, oxidative stress with increased ROS concentrations led to osmotic dysregulation in cultured rat astrocytes, accompanied by changes of cellular morphology and a long-lasting loss of the MI peak [31]. Cerebral hyperammonemia in hepatic encephalopathy is another example for the converse role of osmotically active solutes: the detoxification of ammonia requires glutamine production in glial cells, which leads to a strong decrease of MI to maintain the osmotic intracellular balance [32]. PINK1 deficiency impairs stimulated Ca^2+^ uptake in the mitochondria and leads to intracellular Ca^2+^ overload, which increases the number of ROS with subsequent mitochondria-dependent apoptosis [18], [19]. Therefore, we hypothesize that our MRSI findings reflect counterbalancing of intracellular Ca^2+^ accumulation by MI depletion in astrocytes of PINK1 mutation carriers.

Finally, we found another significant inverse correlation between high GPC and low FDOPA uptake levels over all study subjects. Freely mobile GPC as an important component of the PDE peak in proton-decoupled ^31^P-MRSI is a breakdown product of phosphatidylcholine and of the phospholipid bilayer of neuronal membranes [33]. PDE resonance has been shown to increase linearly in patients with progressive Alzheimer’s disease (AD), but to decrease with aging in control subjects [34]. Moreover, PDE levels were strongly correlated with the number of senile plaques in AD [35]. Therefore, elevated GPC levels in the putamen of our PINK1 patients and their close correlation with local FDOPA uptake loss might indicate increased phospholipid peroxidation and neuronal membrane degradation due to oxidative stress [36]. However, statistical significance disappeared when heterozygote mutation carriers were analyzed alone suggesting that the positive finding is mainly driven by the data outliers of the two homozygous study subjects. Therefore, we believe that the biological significance of our GPC findings is questionable and needs validation in further studies. This holds also true for the small number of individuals with PINK1 mutations who could be measured with combined MRSI so far.

Taken together, our metabolic brain imaging data provide in-vivo evidence for preserved energy supply coming along with osmotic stress in the striatum of PINK1 mutation carriers who had a clear dopaminergic dysfunction in FDOPA PET. Since mitochondrial dysfunction presumably constitutes a converging pathway in patients with different forms of hereditary and sporadic PD, the reported bioenergetic findings in PINK1 patients may also be true in sporadic cases, and this question has to be addressed in future studies.
